# CoV-UniBind: a unified antibody binding database for SARS-CoV-2

**DOI:** 10.1093/bioadv/vbaf328

**Published:** 2026-01-08

**Authors:** Aryan Bhasin, Francesco Saccon, Callum Canavan, Andrew Robson, Joao Euko, Alexandra C Walls, Yunguan Fu

**Affiliations:** Applied AI, InstaDeep Ltd, London, W2 1AY, United Kingdom; Applied AI, InstaDeep Ltd, London, W2 1AY, United Kingdom; Applied AI, InstaDeep Ltd, London, W2 1AY, United Kingdom; Applied AI, InstaDeep Ltd, London, W2 1AY, United Kingdom; Applied AI, InstaDeep Ltd, London, W2 1AY, United Kingdom; Systems Immunology, BioNTech US, Cambridge, MA 02139, United States; Applied AI, InstaDeep Ltd, London, W2 1AY, United Kingdom

## Abstract

**Summary:**

Since the emergence of SARS-CoV-2, numerous studies have investigated antibody interactions with viral variants *in vitro*, and several datasets have been curated to compile available protein structures and experimental measurements. However, existing data remain fragmented, limiting their utility for the development and validation of machine learning models for antibody–antigen interaction prediction. Here, we present CoV-UniBind, a unified database comprising over 75 000 entries of SARS-CoV-2 antibody–antigen sequence, binding, and structural data, integrated and standardized from three public sources and multiple peer-reviewed publications. To demonstrate its utility, we benchmarked multiple protein folding, inverse folding, and language models across tasks relevant to antibody design and vaccine development. We expect CoV-UniBind to facilitate future computational efforts in antibody and vaccine development against SARS-CoV-2.

**Availability and implementation:**

The curated datasets, model scores and antibody synonyms are free to download at https://huggingface.co/datasets/InstaDeepAI/cov-unibind. Folded structures are available upon request.

## 1 Introduction

Since the emergence of the Severe Acute Respiratory Syndrome Coronavirus 2 (SARS-CoV-2), extensive global efforts have focused on the development of vaccines (Ndwandwe and Wiysonge 2021, Titball *et al.* 2024) and monoclonal antibodies (Hwang *et al.* 2022) to combat the COVID-19 pandemic. These initiatives have generated a vast amount of publicly available genomic (Shu and McCauley 2017), structural (Raybould *et al.* 2021), and biochemical data (Harrison *et al.* 2021, Oxford *et al.* 2021, Sarhan *et al.* 2021), providing a valuable foundation for data-driven research. Simultaneously, rapid advancements in artificial intelligence (AI) have enabled the application of deep learning models across multiple areas of vaccine and therapeutic development (El Arab *et al.* 2025), including variant monitoring (Beguir *et al.* 2023), antibody binding prediction (Dreyer *et al.* 2025), epitope identification (Wang *et al.* 2024), and mutational impact on immune recognition (Lv *et al.* 2021). Despite the increased use of AI in SARS-CoV-2 research, systematic evaluation of the models used remains limited. As COVID-19 transitions into a long-term global challenge, a growing need exists for sustained and rigorous assessment of these AI methods. Therefore, a comprehensive benchmarking database is essential to evaluate the utility and performance of these AI tools in COVID-19-related applications over time.

One critical challenge in effective model benchmarking is the fragmented landscape of existing datasets, characterized by inconsistent annotations and limited structural data [only ∼1270 relevant structures are currently available in the RCSB Protein Data Bank (PDB) (Berman *et al.* 2000)]. This limitation presents complications when evaluating protein folding and inverse folding models, which require large amounts of high-quality, annotated structural data. While some databases aim to integrate multiple data sources and standardize benchmarking (Guest *et al.* 2021, Mahita *et al.* 2023, Notin *et al.* 2023), they often suffer from limited experimental coverage, incomplete structural data, or lack of open access. SARS-CoV-2-specific benchmarking resources are particularly scarce. Notably, this challenge is not limited to AI model evaluation but also affects experimental studies, where the lack of standardized benchmarks hampers reproducibility and cross-study comparisons.

To address these limitations, we present CoV-UniBind, a unified antibody binding database for SARS-CoV-2, which integrates high-resolution antibody–antigen complex structures with diverse experimental data capturing complementary aspects of antibody–antigen interactions. These data aim to provide a foundation for understanding SARS-CoV-2 evolution and guiding the design of next-generation vaccines and therapeutic antibodies against emerging variants. CoV-UniBind integrates binding annotations from the Coronavirus Antibody Database (CoV-AbDab) (Raybould *et al.* 2021), providing curated evidence of lineage-specific antibody responses. Precise kinetic measurements of binding strength and interaction dynamics are provided by protein–protein interaction assays, such as surface plasmon resonance (SPR) experiments (Huhn *et al.* 2025). Complementing these data, we include fine-grained, residue-level analyses which assess the impact of individual mutations on antibody binding, from a high-throughput strategy known as deep mutational scanning (DMS) (Greaney *et al.* 2022). In addition to the binding-focused datasets, the Drug Resistance Database (DRDB) (Tzou *et al.* 2022) provides quantitative neutralization potency measurements, offering a comprehensive perspective on antibody efficacy against diverse viral variants. Available structural information in the Structural Antibody Database (SAbDab) (Dunbar *et al.* 2014) can be further processed to derive epitope and paratope information, antibody variable heavy/light chain (VH/VL) sequences and complementarity-determining regions (CDRs). By integrating these diverse yet interlinked data types, CoV-UniBind provides a comprehensive resource for benchmarking and advancing computational models of SARS-CoV-2 antibody–antigen interactions.

To demonstrate the utility of the curated database, we benchmarked a selection of folding (Jumper *et al.* 2021, Boitreaud *et al.* 2024, Wohlwend *et al.* 2024), inverse folding (Dauparas *et al.* 2022, Hsu *et al.* 2022, Dreyer *et al.* 2023, Goverde *et al.* 2024, Høie *et al.* 2025, Shuai *et al.* 2025), and protein language models (PLMs) (Kenlay *et al.* 2024, Olsen *et al.* 2024) on antibody–antigen binding ranking across two categories: ranking multiple antigen variants for each antibody and ranking different antibodies for each antigen variant. These tasks reflect critical applications in vaccine development and therapeutic antibody design, respectively, showcasing the value of CoV-UniBind.

## 2 Methods

### 2.1 Data sources

The CoV-UniBind database was curated from multiple public sources, including SAbDab (Dunbar *et al.* 2014) for protein structures, CoV-AbDab (Raybould *et al.* 2021) comprising lineage-specific binding and neutralization labels, and the COVID DRDB (Tzou *et al.* 2022) containing half-maximal inhibitory concentration (IC_50_) from virus neutralization assays against monoclonal antibodies. Mutational data for multiple SARS-CoV-2 lineages was obtained from outbreak.info (Gangavarapu *et al.* 2023). In addition, data from several peer-reviewed studies were collected and integrated. Namely, Greaney *et al.* (2022), Cao *et al.* (2023) provided binding escape data from DMS experiments, Huhn *et al.* (2025), Zhang *et al.* (2021), Ju *et al.* (2020) provided kinetic data from SPR assays, and Jian *et al.* (2025) provided binding data (IC_50_) from enzyme-linked immunosorbent assay (ELISA) experiments.

### 2.2 Antibody–antigen structure curation

The antibody–antigen protein structures were retrieved from SAbDab in the Chothia scheme (Al-Lazikani *et al.* 1997), and restricted to entries for which the antigen is the SARS-CoV-2 spike glycoprotein. Water molecules and heteroatoms were removed. Amino acid sequences were additionally obtained from the PDB (Rose *et al.* 2011), used to extract antibody names, VH and VL chain sequences, and CDR amino acid sequences using ANARCI (Dunbar and Deane 2016). The CDR residue ranges used are defined by MacCallum *et al.* (1996). Given that a single PDB structure can contain a full spike trimer with multiple associated antibodies, each complex structure was split into individual antibody–antigen pairs (based on those in contact), with the antibody trimmed to include only the VH and VL domains, and the antigen trimmed to the receptor-binding domain (RBD) or N-terminal domain (NTD). Epitope and paratope residues were identified as contact residues between the CDRs and the antigen. Following Jiang *et al.* (2023), two residues were defined as contacting if the minimum distance between any of their atoms was ≤5Å.

### 2.3 Antibody identification

Antibodies often have synonyms, and annotations can vary across datasets. Therefore, efforts to mitigate such differences are crucial. To standardize naming, we constructed an antibody synonym library from the antibody records in SAbDab. Each antibody can be uniquely identified by its name or six CDR amino acid sequences: CDR H1/2/3, CDR L1/2/3. Antibodies with different VH and VL sequences but identical CDRs are considered functionally equivalent with respect to antigen interaction. Since CoV-AbDab provided VH and VL sequences, antibody matching to structural data from SAbDab was performed by comparing CDRs. For the DMS, SPR and IC_50_ datasets, standardized antibody synonyms and PDB IDs, when available, were used to match the entries with their respective structural information, as the antibody sequences are not available.

### 2.4 Evaluation of models for antibody–antigen interaction prediction

To showcase the uses of CoV-UniBind, we evaluate a set of folding, inverse folding, and language models for prediction of antibody-antigen interactions. This evaluation is split into two primary tasks: antigen ranking against a given antibody and antibody ranking against antigen variants. For both ranking tasks, model performance is evaluated using receiver operating characteristic area under the curve (ROC-AUC) for the CoV-AbDab dataset using binary binding labels and the mean Spearman correlation coefficient (${\bar{\rho }}_{S}\pm \text{SEM}$) for those datasets with continuous values. Performance is aggregated across multiple antibody scores for antigen ranking and across multiple antigens for antibody ranking. To ensure robust analyses, antibody ranking was restricted to antigen lineages with a minimum of 20 associated antibodies, and antigen ranking was evaluated only for antibodies with a minimum of 4 associated antigen lineages. Metric signs were inverted when necessary, such that higher values indicate stronger binding, enabling consistent comparisons of ROC-AUC values and Spearman correlation coefficients. For benchmarking the folding models, we computed model-specific metrics and evaluated each model using its best-performing metric. A two-tailed Mann-Whitney U-test was used to compare the ROC-AUC values of groups of antibodies binding to two different domains of the spike protein.

## 3 Results and discussion

**Figure 1.**
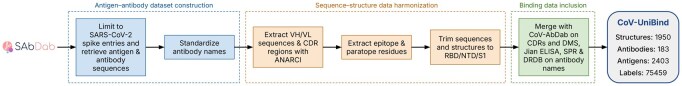
Overview of the data curation workflow for constructing CoV-UniBind. SAbDab entries were restricted to SARS-CoV-2 spike glycoprotein and antibody names were standardized across datasets. ANARCI was used to extract the VH and VL sequences as well as CDR domains from full antibody heavy and light chain sequences. The antigen sequences and structures were trimmed to RBD/NTD/S1 depending on where the antibody binds. Finally, the experimental binding data from the other sources were incorporated, merging on CDR regions or standardized antibody names.

**Figure 2.**
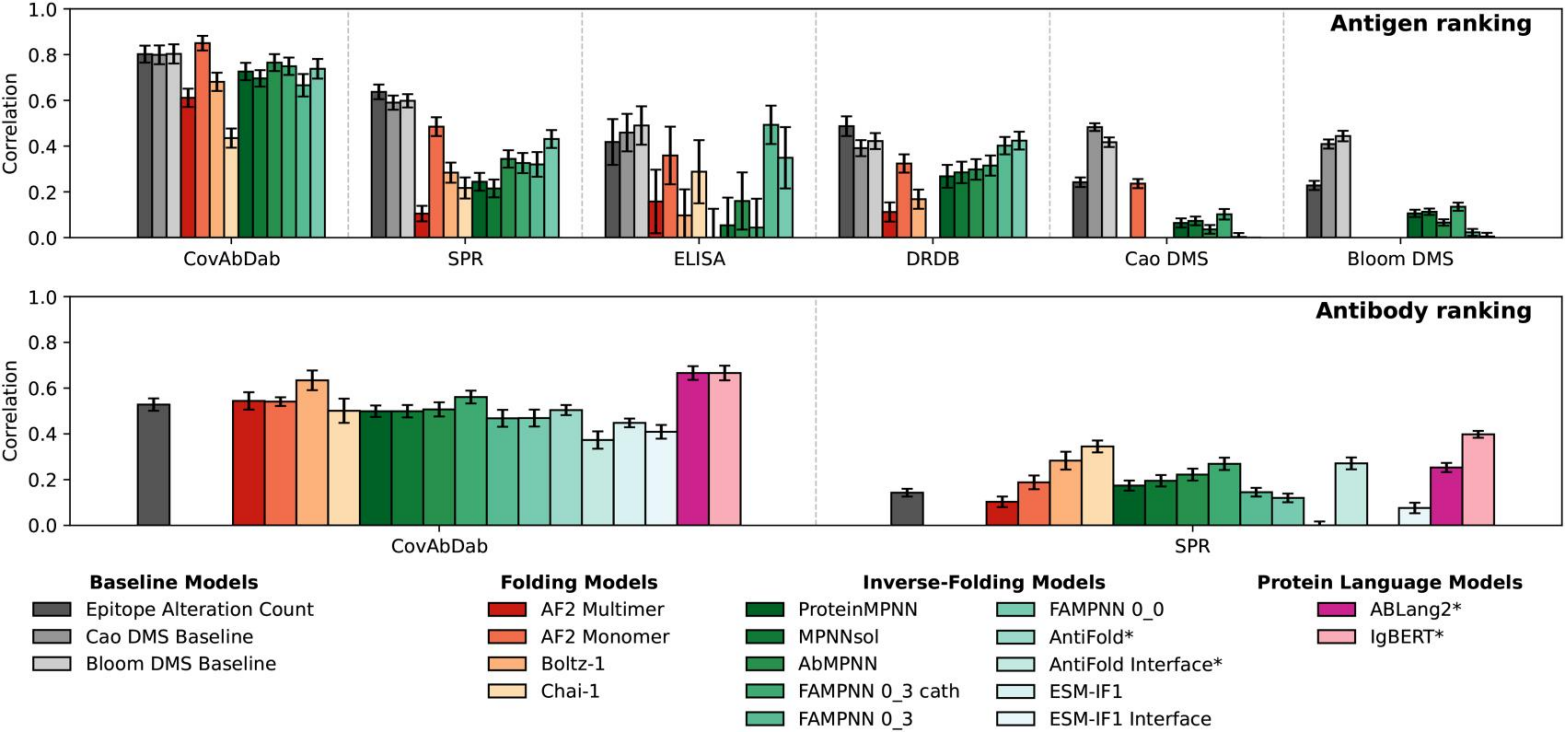
Performance metrics for antigen and antibody ranking across binding datasets and models. Reported values include the mean ± standard error for both ROC-AUC and Spearman correlation coefficient (${\bar{\rho }}_{S}$). Given the relatively weak performance of AF2-multimer, Boltz-1, and Chai-1 on the CoV-AbDab, ELISA, SPR, and DMS-Cao benchmarks, these models were not further evaluated on the DMS-Cao dataset. Likewise, AF2-multimer, AF2-monomer, Boltz-1, and Chai-1 were omitted from the DMS-Bloom evaluation to limit unnecessary computational expenditure. DMS baseline scores were constant across antigen lineages resulting in no correlations across both datasets in the antibody ranking task. Asterisks mark models fine-tuned on antibody sequences/structures.

The CoV-UniBind database was obtained by extracting, integrating and enhancing a diverse set of binding and structural data for antigen-antibody interactions pertaining to SARS-CoV-2 (Fig. 1, Table 1, available as supplementary data at *Bioinformatics Advances* online). The resulting database comprises 1950 antibody–antigen complex structures (processed from 177 unique PDB structures), 73 unique antigen lineages (2403 antigen variants, including 2179 single-point mutants) and 183 unique antibodies. An expanded summary of the composition and characteristics of each data source is presented in Table 1. 96% of the antibodies represented in the datasets bind to the RBD and most of these bind to the ACE2 binding interface, at epitope positions between 437 and 508 (Fig. 1, available as supplementary data at *Bioinformatics Advances* online). Only 6 antibodies (4a8, c1533, cm25, cv3_13, fab4_8 and s2_x333) in the CoV-AbDab dataset bind to the NTD. The database includes one antibody that binds to the S1 C-terminal subdomain (s3h3), whose epitope lies between residues 532 and 619 (Xu *et al.* 2022) The low number of entries corresponding to NTD and S1 binding antibodies, and the lack of S2-binding antibodies, in the database can be attributed to the lack of binding data in CoV-AbDab. The most represented antigen lineages in the database are Omicron variants (BA.1, BA.2, BA.4, BA.5, and XBB) together with wild-type and pre-Omicron variants such as Delta (B.1.617.2), as illustrated in Fig. 2, available as supplementary data at *Bioinformatics Advances* online.

**Table 1.** Summary of antibody–antigen interaction datasets.^a^

Dataset name	Label	Assay	Source	Number of entries	Antibodies	Antigen variants
CoV-AbDab	Binding	Various assays	Raybould *et al.* (2021) (CoV-AbDab)	632	55	40
DRDB	IC_50_ (neutralization)	Various assays	Tzou *et al.* (2022) (DRDB)	2472	89	274
ELISA	IC_50_ (binding)	ELISA	Jian *et al.* (2025)	164	9	38
DMS Cao	Antibody escape	DMS	Cao *et al.* (2023)	9691	85	1684
DMS Bloom	Antibody escape	DMS	Greaney *et al.* (2022)	61 661	89	2145
SPR	$K_{D}$	SPR	Zhang *et al.* (2021), Huhn *et al.* (2025), Ju *et al.* (2020), Huang *et al.* (2022), He *et al.* (2023)	839	80	12
**CoV-UniBind**				75 460	183	2403

a Unique antigen variants include both lineages from outbreak.info and single-point mutants generated in DMS studies.

To benchmark models, we framed CoV-UniBind as two predictive tasks: (i) ranking antigens by predicted binding affinity for each antibody, and (ii) ranking antibodies for each antigen. This formulation addresses dataset limitations (e.g. DMS data are normalized per antibody) and supports distinct applications: antigen ranking aids vaccine design by assessing escape potential, while antibody ranking supports therapeutic discovery by identifying potent binders. The results are summarized in Fig. 2.

CoV-AbDab provides binary binding labels for a diverse set of antibodies. The nature of this dataset makes it well-suited for antigen (or antibody) classification tasks. In the antigen ranking task, all models demonstrate moderate to good performance (ROC-AUC ≥ 0.435) on the CoV-AbDab dataset (Table 2, available as supplementary data at *Bioinformatics Advances* online), where the AF2-monomer model with gap trick achieves the highest mean ROC-AUC of 0.850±0.032. In contrast, all tested deep learning models struggled to generalize to the DMS datasets, with mean Spearman correlations ${\bar{\rho }}_{S}\leq 0.236$. This underscores a key challenge posed by the DMS data, which consist of single-point mutations, where escape is measured indirectly as a proxy for binding affinity (Chi *et al.* 2024). The ELISA and SPR datasets, like CoV-AbDab, involve comparisons across antibodies and viral lineages (including multiple mutations) and offer more direct measures of binding strength, either via potency (IC_50_) values or kinetic (k_D_) data. Inverse folding models, particularly FAMPNN, exhibit strong correlations on these datasets, achieving a mean Spearman correlation of ${\bar{\rho }}_{S}=0.493$ on the ELISA dataset. The diversity of antibody binding sites in CoV-AbDab, including 49 targeting the RBD and 6 targeting the NTD, also allows for benchmarking antigen ranking models across both RBD and NTD subsets (Table 3, available as supplementary data at *Bioinformatics Advances* online).

Table 4, available as supplementary data at *Bioinformatics Advances* online summarize benchmarking results for the antibody ranking task. Alongside structural models, antibody-specific PLMs were assessed, owing to their strong performance in zero-shot antibody-antigen binding predictions (Kenlay *et al.* 2024, Høie *et al.* 2025). The performance of these models is generally superior to that of other deep learning models, with IgBERT and AbLang2 demonstrating the strongest predictive performance on the CoV-AbDab benchmark (ROC-AUC = 0.666±0.032 and 0.666±0.030, respectively), highlighting the benefit of pretraining on antibody sequences. IgBERT additionally achieves the highest correlation on the SPR dataset (${\bar{\rho }}_{S}=0.398\pm 0.015$), while Chai-1 ranks second best on SPR (${\bar{\rho }}_{S}=0.345\pm 0.026$). Notably, we observe a drop in performance of models and baselines in the antibody ranking tasks compared to antigen ranking tasks (e.g. 36% drop of AF2 monomer model performance on CoV-AbDab). This discrepancy likely stems from both biological and dataset-level factors. Antibody variable domains are highly flexible and structurally diverse, whereas antigenic regions like the RBD and NTD are comparatively rigid and easier to model. Importantly, antigen ranking involves mutations at a fixed epitope (the antibody’s binding site), while antibody ranking involves multiple epitopes across the antigen surface. Consequently, mutation-based predictors lose explanatory power when multiple biological factors affect binding, leading to weaker correlations.

Beyond predicting binding affinity, we explored the ability of our models to predict the neutralization potency of individual antibodies across a broad spectrum of viral variants using the DRDB dataset (see Table 5, available as supplementary data at *Bioinformatics Advances* online), a task that reflects a more complex and biologically downstream effect than binding alone. It is important to note that IC_50_ values in DRDB stem from diverse assay conditions, such as lentivirus and HIV-based pseudovirus systems, which introduce variability and limit direct comparability across antibodies. Encouragingly, FAMPNN checkpoints performed competitively, especially when compared to structure-based folding models, achieving a mean ${\bar{\rho }}_{S}\approx 0.38$ across checkpoints. Notably, a simple baseline based on epitope alteration counts achieved even stronger performance, with a correlation of 0.487±0.043, highlighting both the challenge and the opportunity for further model development in this domain.

## 4 Conclusions and outlooks

CoV-UniBind was developed as a centralized, accessible and comprehensive resource for structural binding and neutralization data of antibodies targeting SARS-CoV-2 and its variants. By curating and integrating data from a diverse array of sources, the dataset offers a rich and multifaceted view of antibody–antigen interactions. This heterogeneity enables the representation of a broad spectrum of binding affinities, neutralizing potencies, and structural variations, which are often underrepresented in individual datasets. Importantly, we demonstrated that this diversity provides a strong foundation for benchmarking predictive models, enabling the evaluation of their performance across a wide range of biological and experimental contexts.

Our benchmarking results indicate that both folding and inverse folding models can show strong performance, depending on the task, suggesting that both approaches should be considered for structure-based antibody–antigen binding prediction. Performance varied across datasets, highlighting the need for task-specific fine-tuning. Despite promising results on the datasets, considerable room for improvement remains, particularly for inverse folding models. The fine-tuned antibody-specific PLMs performed remarkably well on the antibody-ranking task, particularly impressive given that no antigen information was provided during inference. The lightweight nature of these models underscores the ability of PLMs to extract high-level functional signals directly from sequence alone, making them effective for ranking antibodies against a fixed antigen when data is available. In parallel, our findings indicate that simple baseline models can provide surprisingly informative predictions. This highlights the importance of including a range of model types in benchmarking efforts, as even minimal approaches offer valuable context for evaluating and interpreting the performance of more complex methods.

As new SARS-CoV-2 variants continue to emerge (e.g. LP.8.1 and NB.1.8.1, designated by the World Health Organization as variants under monitoring on 24 January 2025 and 23 May 2025, respectively), carrying mutations with potential implications for immune evasion and reduced vaccine efficacy (Guo *et al.* 2025, Liu *et al.* 2025), there is a growing need for robust tools to anticipate and mitigate such threats. We anticipate that CoV-UniBind will serve as a valuable resource for benchmarking predictive models and as a versatile platform to support the rational design of next-generation antibodies and vaccines in response to ongoing viral evolution (Goodswen *et al.* 2023, Williams *et al.* 2023, Dreyer *et al.* 2025). By offering a comprehensive, integrated view of antibody–antigen interactions across diverse structural and functional landscapes, CoV-UniBind lays the groundwork for accelerating therapeutic development in response to SARS-CoV-2 evolution. Moreover, the framework established here can be readily adapted to future viral threats, contributing to a more agile and informed pandemic preparedness strategy.

## Supplementary Material

vbaf328_Supplementary_Data

## Data Availability

The curated datasets, model scores and antibody synonyms are free to download at https://huggingface.co/datasets/InstaDeepAI/cov-unibind. Folded structures are available upon request.
