# Supplementary material for: CoV-UniBind: a unified antibody binding database for SARS-CoV-2
Source: Bioinform Adv. 2026 Jan 8;6(1):vbaf328. doi: 10.1093/bioadv/vbaf328 (PMC12800777; doi:10.1093/bioadv/vbaf328)
Supplement: vbaf328_Supplementary_Data [file vbaf328_supplementary_data.pdf]

Supplementary Information

Antibody–antigen interaction prediction

AlphaFold2 (AF2) was evaluated in two different modes: multimer model and monomer model with gap trick [Bryant et al., 2022]. The predicted local distance difference test (plDDT), predicted aligned error (PAE), and predicted template modelling (pTM) metrics were extracted from the predictions, where larger plDDT and pTM values, and lower PAE values, are intuitively hypothesized to correlate with stronger binding affinity. Both configurations were run with default parameters, without multiple sequence alignment (MSA), and using a custom structural template, a complex of the antibody with a reference antigen. For each antibody, a single custom template was reused across all associated variants. The `model_2_ptm` checkpoint was used for all predictions. Boltz-1 and Chai-1 were evaluated similarly, with predicted docking error (PDE) instead of PAE for Boltz-1, in addition to the other metrics [Wohlwend et al., 2025, Boitreaud et al., 2024]. It was executed with its default settings, also without the use of templates or MSA. All folding experiments (using AF2-Monomer with gap trick, AF2-Multimer, Boltz-1, and Chai-1) were run with an internal folding pipeline, which enables fast and high-throughput protein folding predictions via the Google Cloud Platform (GCP) environment. The metric from each folding model that produced the best performance was plotted in Figure 2. Ranking correlations for all the folding model metrics are shown in Figure S3.

For inverse folding models (protein message passing neural networks (MPNN) [Gilmer et al., 2017], MPNNsol [Goverde et al., 2024], AbMPNN [Dreyer et al., 2023], and FAMPNN [Shuai et al., 2025]), the binding is estimated using the log-likelihood of the introduced antigen mutations with respect to a reference antigen sequence. Scoring was conditioned on the structure of an antigen–antibody complex, typically using the wild-type antigen as the structural reference. For FAMPNN, 10 sampling runs were performed with different random seeds, all with a batch size of 16, and the final score of each antigen was computed as the mean across these 10 samples. Three model checkpoints were tested, namely `0_0`, `0_3` and `0_3_cath` as described in Shuai et al. [2025]. For other inverse folding models, 100 sequence samples were generated per input, with the final score defined as the mean across those 100 samples. A batch size of 100 and a fixed random seed of 42 were used. For model benchmarking, we used the negative likelihood ratio from FAMPNN and the negative log-likelihood scores from the backbone-only inverse folding models (ProteinMPNN, MPNNsol, and AbMPNN).

As additional baselines, we included two non-deep learning methods. The first is epitope alteration count [Beguir et al., 2023], which calculates the number of antigen mutations occurring within the structure-derived epitope. A higher count is expected to correspond to reduced binding, reflecting the assumption that more alterations in the binding interface lead to binding abrogation. This approach does not consider the biochemical nature or positional impact of the mutations. The second baseline leverages DMS data. For each single-point mutation, the escape value was first averaged across all antibodies in the corresponding DMS dataset. These values were then summed to produce a final score for multi-point mutants, with higher scores indicating greater escape potential and thus lower expected binding. This procedure was applied separately using the Bloom and Cao DMS datasets, resulting in two baselines: Bloom-based and Cao-based DMS scores.

In addition to benchmarking folding and inverse-folding models, we evaluated protein language models (PLMs) for antibody ranking. Antibody-specific models IgBERT [Kenlay et al., 2024] and AbLang2 [Olsen et al., 2024] were used to generate sequence-level embeddings for each antibody. The evaluation was restricted to antigen lineages with at least 20 associated antibodies (CoV-AbDab, $n = 16$; SPR, $n = 12$). For each lineage, paired sequence embeddings were extracted for the corresponding antibodies (excluding antigen sequences) and used to train a Ridge regression model with 5-fold cross-validation. Subsequently, the fitted model was used to predict binding values per antibody–antigen pair. The inverse-folding model ESM-IF1 [Hsu et al., 2022] and the fine-tuned version on antibody structures, AntiFold [Høie et al., 2025], were similarly evaluated on the same lineage subset. For each antibody–antigen complex, the model generated residue-level logits across all amino acids, which were summed over the native residues to obtain a sequence-level score. Since each PDB template contained a single antigen variant per antibody, changes in logit scores due to introduced mutations were used to derive updated scores for each antigen-variant:antibody complex. Two metrics were computed per complex, the global sum of logits across all residues and the epitope-localized sum of logits (reported as "AntiFold/ESM-IF1 Interface" in Table S4), and used for the antibody ranking task as above.

Figures & tables

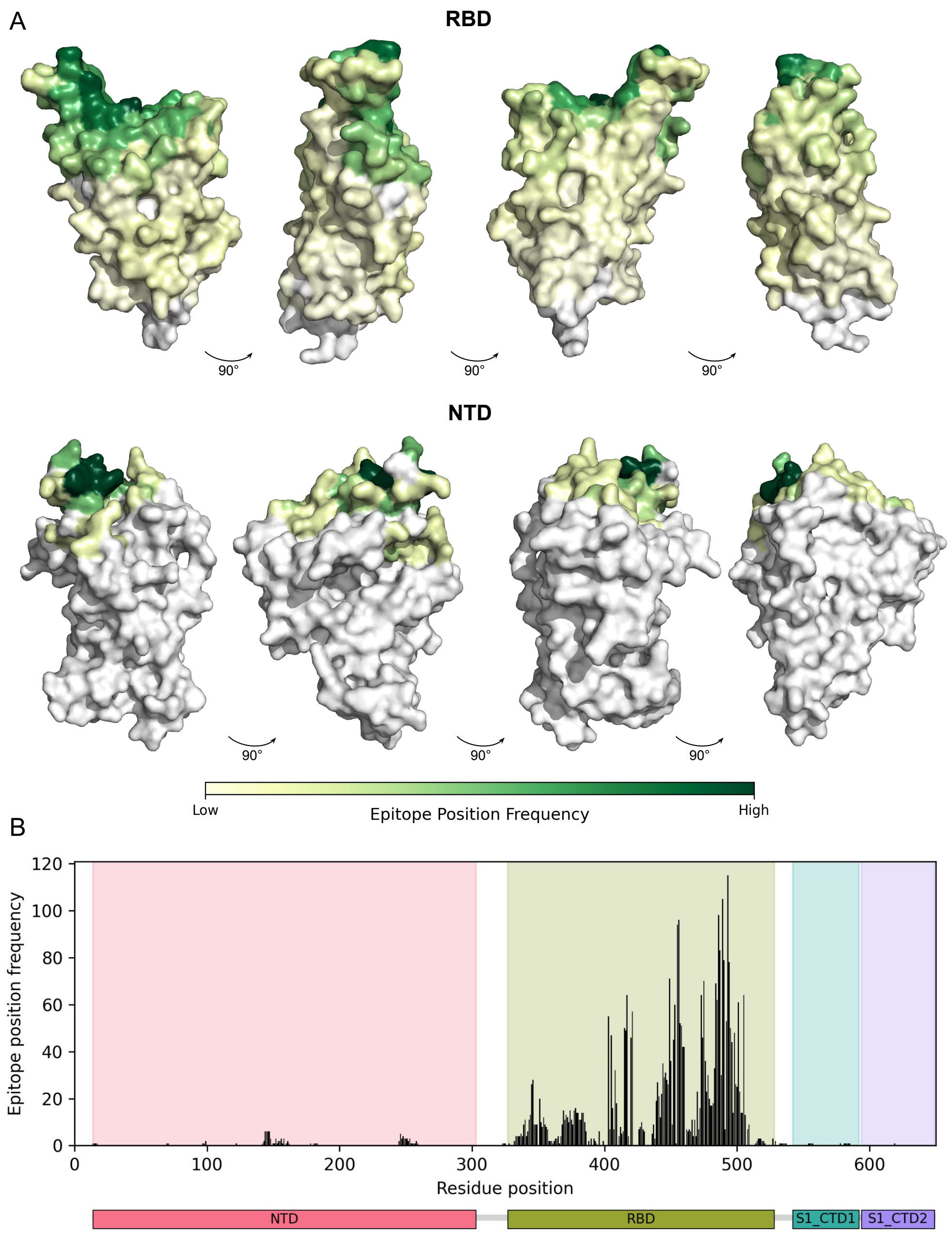

Figure S1. Hotspots of epitope residues on the spike RBD and NTD domains from the total pool of antibodies in CoV-UniBind. Darker green corresponds to a higher frequency of the epitope residue. In white, residues not in contact with antibodies in the database. (B) Frequency of the positions in the epitopes. Epitope domains are shown as shaded areas and marked below the plot.

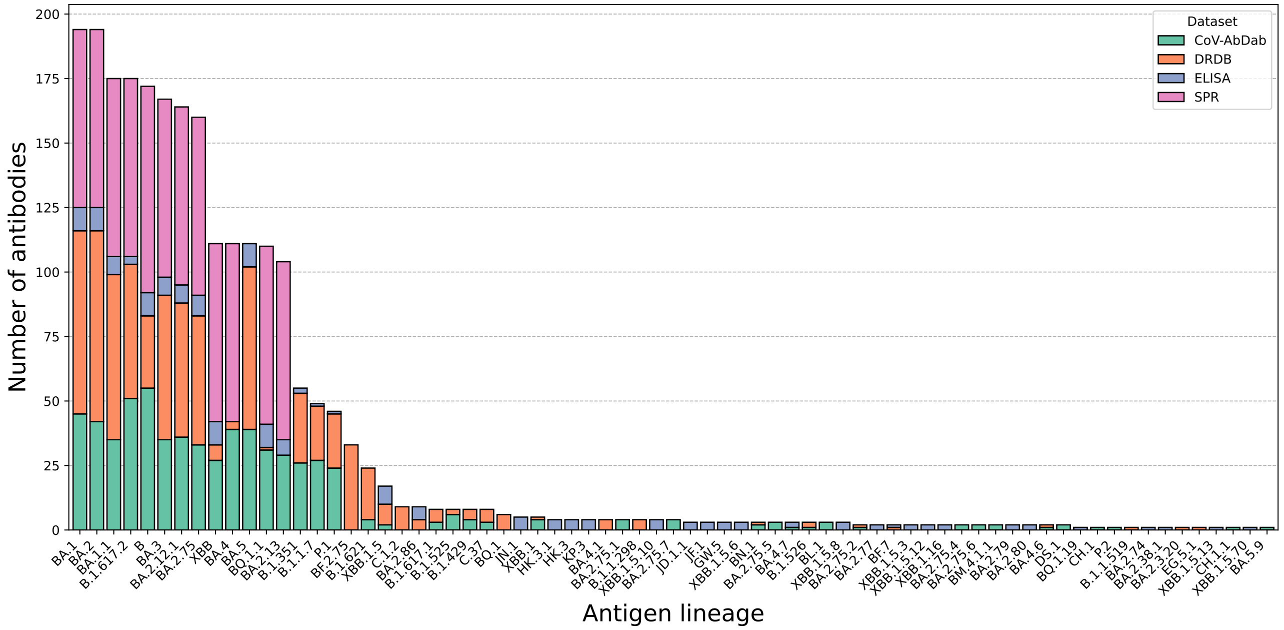

Figure S2. Representation of antibody coverage across antigen lineages in the CoV-AbDab, DRDB, ELISA, and SPR datasets. Each bar shows the total number of antibodies for a given lineage, with segments indicating contributions from different data sources.

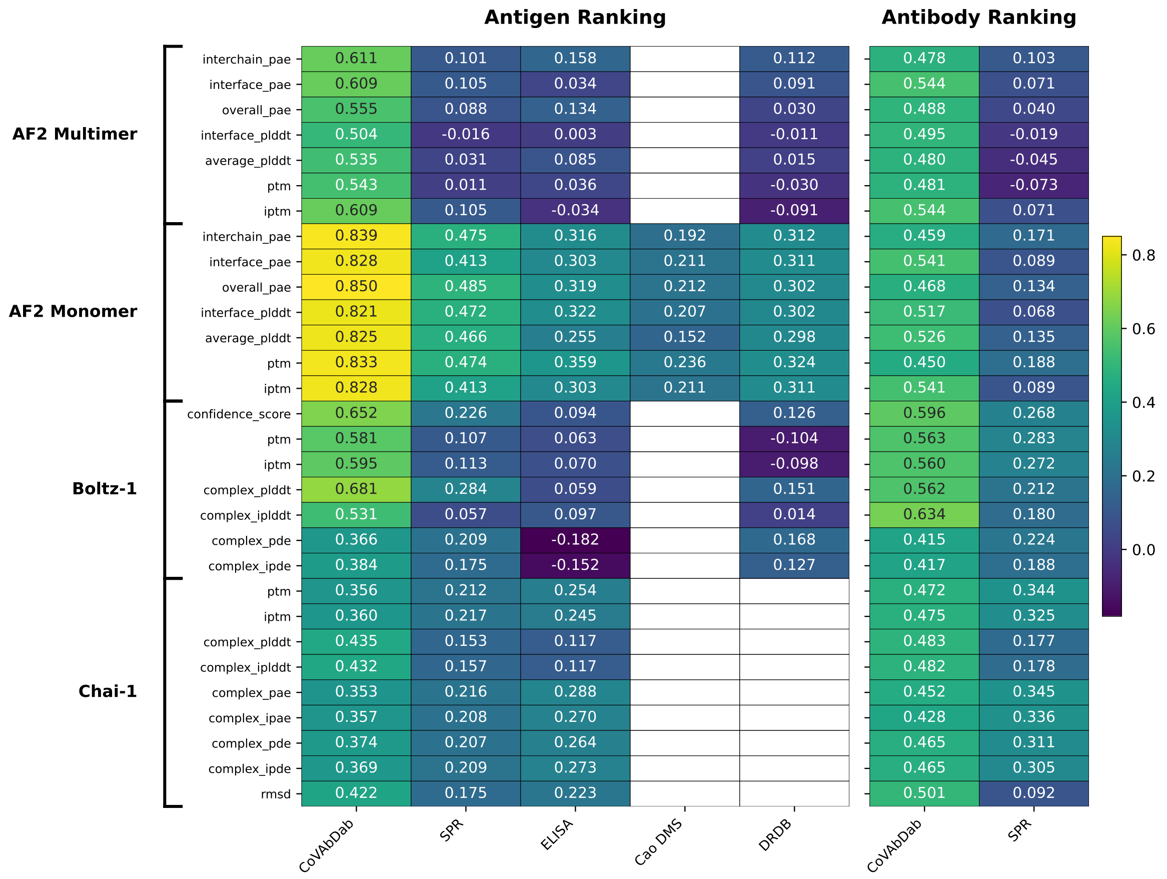

Figure S3. Average performance metrics (ROC-AUC or Spearman correlation coefficients, $\overline{\rho}_S$) for antigen and antibody ranking tasks calculated for all folding model metrics.

Dataset	Binding/Neutralisation Data	PDB IDs	Ab/Ag Chain IDs	VH–VL sequences	Epitopes/Paratopes	Trimmed/Processed Structures	Final update
CoV-AbDab	✓	✓					08/02/2024
DRDB	✓						13/05/2024
ELISA	✓						01/2025
DMS Cao	✓						19/12/2022
DMS Bloom	✓						11/05/2022
SPR	✓						02/01/2025
SAbDab		✓	✓				Continuous
**CoV-UniBind**	✓	✓	✓	✓	✓	✓	04/04/2025

Table S1. Overview of how CoV-UniBind integrates information from multiple data sources (CoV-AbDab, DRDB, ELISA, DMS Cao, DMS Bloom, SPR and SAbDab). Each row represents a data source and each column a data aspect. Check marks indicate which aspects are present in each source. CoV-UniBind combines all aspects into a standardized and unified resource.

Model	CoV-AbDab Binding ROC-AUC	DMS Cao Antibody Escape $\overline{\rho}_S$	DMS Bloom Antibody Escape $\overline{\rho}_S$	ELISA Binding Potency $\overline{\rho}_S$	SPR Binding Affinity $\overline{\rho}_S$
Epitope Alteration Count	0.802 ± 0.037	0.242 ± 0.021	0.228 ± 0.020	0.418 ± 0.100	**0.637 ± 0.032**
Cao DMS Baseline	0.799 ± 0.041	**0.483 ± 0.017**	<u>0.409 ± 0.020</u>	0.459 ± 0.082	0.590 ± 0.031
Bloom DMS Baseline	<u>0.803 ± 0.042</u>	<u>0.417 ± 0.021</u>	**0.444 ± 0.023**	<u>0.490 ± 0.084</u>	<u>0.598 ± 0.029</u>
AF2 Multimer	0.611 ± 0.040	–	–	0.158 ± 0.139	0.105 ± 0.034
AF2 Monomer	**0.850 ± 0.032**	0.236 ± 0.020	–	0.359 ± 0.126	0.485 ± 0.041
Boltz-1	0.681 ± 0.04	–	–	0.097 ± 0.114	0.284 ± 0.044
Chai-1	0.435 ± 0.042	–	–	0.288 ± 0.138	0.217 ± 0.046
ProteinMPNN	0.726 ± 0.038	0.064 ± 0.020	0.106 ± 0.016	-0.017 ± 0.143	0.244 ± 0.039
MPNNsol	0.696 ± 0.036	0.073 ± 0.019	0.113 ± 0.014	0.054 ± 0.121	0.215 ± 0.039
AbMPNN	0.765 ± 0.037	0.036 ± 0.019	0.066 ± 0.014	0.160 ± 0.125	0.344 ± 0.038
FAMPNN 0_3_cath	0.749 ± 0.038	0.102 ± 0.023	0.135 ± 0.018	0.044 ± 0.126	0.326 ± 0.044
FAMPNN 0_3	0.666 ± 0.049	0.003 ± 0.017	0.023 ± 0.015	**0.493 ± 0.084**	0.320 ± 0.054
FAMPNN 0_0	0.738 ± 0.043	-0.052 ± 0.020	0.005 ± 0.016	0.349 ± 0.134	0.431 ± 0.039

Table S2. Performance metrics for antigen ranking across binding datasets and models. Reported values include the mean ± SEM for both ROC-AUC and Spearman correlation coefficient ($\overline{\rho}_S$). Higher positive values indicate stronger performance, where the best-performing model is highlighted in bold and second best is underlined. – indicates data are not available.

Model	CoV-AbDab Binding (RBD) ROC-AUC	CoV-AbDab Binding (NTD) ROC-AUC
Epitope Alteration Count	0.803 ± 0.040	**0.795 ± 0.069**
Cao DMS Baseline	0.834 ± 0.041	0.513 ± 0.124*
Bloom DMS Baseline	<u>0.840 ± 0.041</u>	0.499 ± 0.124*
AF2 Multimer	0.598 ± 0.041	0.717 ± 0.16
AF2 Monomer	**0.860 ± 0.032**	<u>0.765 ± 0.118</u>
Boltz-1	0.693 ± 0.040	0.589 ± 0.140
Chai-1	0.461 ± 0.046	0.223 ± 0.057
ProteinMPNN	0.736 ± 0.040	0.640 ± 0.129
MPNNsol	0.703 ± 0.039	0.640 ± 0.107
AbMPNN	0.779 ± 0.040	0.654 ± 0.097
FAMPNN 0_3_cath	0.778 ± 0.037	0.515 ± 0.130
FAMPNN 0_3	0.668 ± 0.053	0.644 ± 0.115
FAMPNN 0_0	0.756 ± 0.044	0.593 ± 0.147

Table S3. Performance of binding classification for the antigen ranking task for the Cov-AbDab Lineage-Antibody Binding dataset, split into RBD-targeting and NTD-targeting antibody subsets. Higher positive values indicate stronger performance, where the best-performing model is highlighted in bold and second best is underlined. A Mann-Whitney U-test was performed to test statistical significance; * indicates $p < 0.05$.

Model	CoV-AbDab Binding ROC-AUC	SPR Data $\overline{\rho}_S$
Epitope Alteration Count	0.528 ± 0.027	0.143 ± 0.017
Cao DMS Baseline	–	–
Bloom DMS Baseline	–	–
AF2 Multimer	0.544 ± 0.038	0.103 ± 0.023
AF2 Monomer	0.541 ± 0.019	0.188 ± 0.030
Boltz-1	0.634 ± 0.043	0.283 ± 0.039
Chai-1	0.501 ± 0.053	<u>0.345 ± 0.026</u>
ProteinMPNN	0.499 ± 0.025	0.174 ± 0.022
MPNNsol	0.499 ± 0.027	0.195 ± 0.025
AbMPNN	0.507 ± 0.031	0.222 ± 0.026
FAMPNN 0_3_cath	0.561 ± 0.028	0.269 ± 0.027
FAMPNN 0_3	0.468 ± 0.037	0.145 ± 0.019
FAMPNN 0_0	0.469 ± 0.037	0.120 ± 0.019
IgBERT	<u>0.666 ± 0.032</u>	**0.398 ± 0.015**
AbLang2	**0.666 ± 0.030**	0.253 ± 0.020
AntiFold	0.504 ± 0.022	-0.005 ± 0.022
AntiFold Interface	0.373 ± 0.038	0.271 ± 0.026
ESM-IF1	0.448 ± 0.019	-0.061 ± 0.024
ESM-IF1 Interface	0.409 ± 0.030	0.076 ± 0.023

Table S4. Performance metrics for antibody ranking across datasets and models. Reported values include the mean ± SEM for both ROC-AUC and Spearman correlation coefficient ($\overline{\rho}_S$). Higher positive values indicate stronger performance, where the best-performing model is highlighted in bold and the second best-performing model is underlined. – indicates where data are not available. Antibody ranking was performed on antigen lineages with 20 or more associated antibody entries.

Model	DRDB Neutralisation Potency $\overline{\rho}_S$
Epitope Alteration Count	**0.487 ± 0.043**
Cao DMS Baseline	0.391 ± 0.035
Bloom DMS Baseline	0.422 ± 0.035
AF2 Multimer	0.112 ± 0.042
AF2 Monomer	0.324 ± 0.040
Boltz-1	0.168 ± 0.042
ProteinMPNN	0.268 ± 0.050
MPNNsol	0.285 ± 0.047
AbMPNN	0.298 ± 0.045
FAMPNN 0_3 cath	0.315 ± 0.044
FAMPNN 0_3	0.402 ± 0.038
FAMPNN 0_0	<u>0.424 ± 0.039</u>

Table S5. Performance metrics for antigen ranking using neutralization IC_{50} data from DRDB. Reported values include the mean ± SEM for Spearman correlation coefficients ($\overline{\rho}_S$). Higher positive values indicate stronger performance, where the best-performing model is highlighted in bold and second-best is underlined. Chai-1 was excluded due to its relatively low performance in antigen ranking on the CoV-AbDab, ELISA and SPR datasets. PLMs were excluded as they do not take antigen information as input.

References

K. Beguir, M. J. Skwark, Y. Fu, T. Pierrot, N. L. Carranza, A. Laterre, I. Kadri, A. Korched, A. U. Lowegard, B. G. Lui, et al. Early computational detection of potential high-risk sars-cov-2 variants. Computers in biology and medicine, 155:106618, 2023.

J. Boitreaud, J. Dent, M. McPartlon, J. Meier, V. Reis, A. Rogozhnikov, and K. Wu. Chai-1: Decoding the molecular interactions of life. BioRxiv, 2024.

P. Bryant, G. Pozzati, and A. Elofsson. Improved prediction of protein-protein interactions using alphafold2. Nature Communications, 13(1):1265, 2022. doi:10.1038/s41467-022-28865-w. URL https://doi.org/10.1038/s41467-022-28865-w.

F. A. Dreyer, D. Cutting, C. Schneider, H. Kenlay, and C. M. Deane. Inverse folding for antibody sequence design using deep learning. 2023 ICML Workshop on Computational Biology, 2023. URL https://arxiv.org/abs/2310.19513.

J. Gilmer, S. S. Schoenholz, P. F. Riley, O. Vinyals, and G. E. Dahl. Neural message passing for quantum chemistry. In International conference on machine learning, pages 1263–1272. PMLR, 2017.

C. A. Goverde, M. Pacesa, N. Goldbach, L. J. Dornfeld, P. E. M. Balbi, S. Georgeon, S. Rosset, S. Kapoor, J. Choudhury, J. Dauparas, C. Schellhaas, S. Kozlov, D. Baker, S. Ovchinnikov, A. J. Vecchio, and B. E. Correia. Computational design of soluble and functional membrane protein analogues. Nature, 631(8020):449–458, July 2024. ISSN 1476-4687. doi:10.1038/s41586-024-07601-y. URL https://doi.org/10.1038/s41586-024-07601-y.

M. H. Høie, A. M. Hummer, T. H. Olsen, B. Aguilar-Sanjuan, M. Nielsen, and C. M. Deane. Antifold: Improved structure-based antibody design using inverse folding. Bioinformatics Advances, 5(1):vbae202, 2025.

C. Hsu, R. Verkuil, J. Liu, Z. Lin, B. Hie, T. Sercu, A. Lerer, and A. Rives. Learning inverse folding from millions of predicted structures. In International conference on machine learning, pages 8946–8970. PMLR, 2022.

H. Kenlay, F. A. Dreyer, A. Kovaltsuk, D. Miketa, D. Pires, and C. M. Deane. Large scale paired antibody language models. PLOS Computational Biology, 20(12):e1012646, 2024.

T. H. Olsen, I. H. Moal, and C. M. Deane. Addressing the antibody germline bias and its effect on language models for improved antibody design. Bioinformatics, 40(11):btae618, 2024.

R. W. Shuai, T. Widatalla, P.-S. Huang, and B. L. Hie. Sidechain conditioning and modeling for full-atom protein sequence design with fampnn. bioRxiv, 2025. doi:10.1101/2025.02.13.637498. URL https://www.biorxiv.org/content/early/2025/02/17/2025.02.13.637498.

J. Wohlwend, G. Corso, S. Passaro, N. Getz, M. Reveiz, K. Leidal, W. Swiderski, L. Atkinson, T. Portnoi, I. Chinn, J. Silterra, T. Jaakkola, and R. Barzilay. Boltz-1 democratizing biomolecular interaction modeling. bioRxiv, 2025. doi:10.1101/2024.11.19.624167. URL https://www.biorxiv.org/content/early/2025/05/06/2024.11.19.624167.
